# Correlation of preferentially expressed antigen of melanoma (PRAME) gene expression with clinical characteristics in acute leukemia patients

**DOI:** 10.1186/s43141-022-00376-7

**Published:** 2022-07-05

**Authors:** Nagaraj V. Kulkarni, Reshma A. Shetty, Suchetha Kumari N, Vijith V. Shetty, Rajesh Krishna, Meenakshi Arumugam, Akanksha A. Kalal, Prashanth Shetty

**Affiliations:** 1 Central Research Laboratory, KSHEMA Centre for Genetic Services, KS Hegde Medical Academy, NITTE (Deemed to be) University, Derlakatte, Mangalore, Karnataka 575 018 India; 2 Department of Biochemistry, KS Hegde Medical Academy, NITTE (Deemed to be) University, Nityanandanagar, Deralakatte, Mangalore, Karnataka 575 018 India; 3 Department of Medical Oncology, KS Hegde Medical Academy, NITTE (Deemed to be University), Derlakatte, Mangalore, Karnataka 575 018 India; 4 Yenepoya Medical College Hospital, Yenepoya (Deemed to be) University, Nityanandanagar, Deralakatte, Mangalore, Karnataka 575 018 India

**Keywords:** Preferentially expressed antigen of melanoma (PRAME), Acute leukemia, Cytogenetics, Reverse transcriptase-quantitative polymerase chain reaction (RT-qPCR)

## Abstract

**Background:**

Preferentially expressed antigen of melanoma (PRAME) gene is regularly overexpressed in acute leukemia (AL) and other malignant diseases which are recognized by human leucocyte antigen (HLA-24) located in the human chromosome of 22q11 coded by 509 amino acids. To rule out the PRAME gene expression in AL patients and its correlation with clinical characteristics in the Indian population set up by RT-qPCR.

**Results:**

A total of 42 samples collected, 29 (69.4%) were males, and 13 (30.95%) were females, with a mean and standard deviation for age were 39.07 ± 22.22 years. Of which AML were of 22 (52.38%) cases, ALL were of 14 (33.33%) cases, and 6 (14.2%) cases which included other forms of leukemia. PRAME gene expression was highly expressed in thirty-three 27 (64.28%) AL patients compared to the least expression in healthy individuals. No significant difference between the different forms of AL (*p*=0.3203) was observed. Cytogenetic analysis of normal karyotype (NK), abnormal karyotype (Ab. K), and culture failure (CF) displayed statistical non-significance (*p*=0.5801). Among cytogenetic abnormalities obtained, no significant differences between the groups were observed (*p*=0.8507). Chloride, potassium, and absolute lymphocyte count (ALC) was found to be statistically significant with *p*=0.0038**, *p*=0.0358*, and *p*=0.0216*, respectively, between all other clinical characteristics. There was no correlation between the PRAME gene expression and clinical parameters.

**Conclusion:**

PRAME gene expression in AL patients was highly expressed, comparable to studies reported globally with significant cytogenetic results. PRAME gene could be used as a potential diagnostic marker for monitoring the malignancies and minimal residual disease in AL.

## Background

Acute leukemia (AL) is a neoplastic disease that occurs by the growth of abnormal lymphoid and myeloid cells. Acute myeloid leukemia (AML) is the most common leukemia in adults, with 80% of cases. The incidence of AML in the USA is 3 to 5 per 100,000 populations [1]. Disturbance in myeloid progenitor cells needs proliferation and clonal expansion of blast. This blast infiltration in the peripheral blood leads to an increased number of non-function immature leukocytes [2]. Acute lymphoblastic leukemia (ALL) is a malignant disorder of lymphoid progenitor cells that affects both adults and children aged 2–5 years, which is one of the frequent hematological malignancies seen in pediatric cases [3]. Approximately, 25 to 40% of childhood cancer in India leads to overall leukemia, in which 60 to 80% are ALL among all leukemia reported cases [4].

The preferentially expressed antigen of melanoma (PRAME) gene is recognized by human leucocyte antigen (HLA-24) located in the human chromosome of 22q11 [5]. Autologous cytolytic T lymphocytes recognize human tumor antigens (CTL), identified by autologous CTL clones from mixed lymphocytic cell cultures, proposed as a potential marker for tumor immunotherapy. PRAME is a gene encoding an HLA-24-restricted antigenic peptide presented to autologous melanoma protein. It encodes a protein of 509 amino acids [6]. PRAME expression levels are generally seen in testis, adrenal, ovary, and endometrium tissues. It has been expressed in various cancers such as malignant melanomas, sarcomas, lung, renal, head, and neck cancers. As reported in international studies, the PRAME gene is also expressed in AML, ALL, multiple myeloma, and chronic myeloid leukemia [7].

The anti-apoptotic proteins encoding for overexpression of genes result in the survival of leukemic cells, which are correlated with relapse and remission of leukemia by controlling the apoptotic mechanisms failing therapy [8]. The mechanism, physiological role, and regulation of the PRAME gene is not clearly understood; hence, the molecular part of the gene is not well discovered. The fusion proteins in AML like Breakpoint Cluster Region-Abelson Murine Leukemia (BCR-ABL) and Acute Myeloid Leukemia-Eight Twenty-One (AML-ETO) have a significant role in the upregulation of the gene associated with AML cases [9]. The PRAME gene dominates the retinoic acid receptor (RAR) signaling pathway, which induces apoptosis, cell differentiation, and proliferation, which are dominated by the PRAME gene, acting as a repressor of RAR signaling pathway. The limitation of RAR signaling is compromised in cancers leading to tumor-suppressive mechanisms. The Promyelocytic leukemia zinc finger- retinoic acid receptor alpha (PLZF-RARα) and Promyelocytic leukemia-retinoic acid receptor alpha (PML-RARα) are the chimeric genes formed from the translocations of RARα in acute promyelocytic leukemia (APML) preventing cell differentiation by altering the receptors which act as vital repressors of transcription leading to cancer [10, 11].

The function of the PRAME gene in leukemic cells seems to be controversial and needs to be explored. PRAME gene expression in acute leukemia has not been studied in the Indian population in correlation with conventional cytogenetics. Globally, many studies have been reported on PRAME gene expression in acute leukemia. Still, in the current study, we have correlated the cytogenetics and clinical data with the mRNA expression of the PRAME gene.

## Methods

### Study setting and patients

This descriptive study was carried out on 42 clinically diagnosed acute leukemia patients from 2018 to 2021, ranging from 1 to 88 years. Twenty-one healthy volunteers were enrolled as control subjects. The study was carried out in Molecular Genetics Laboratory, Central Research Laboratory (KS Hegde Medical Academy, Mangalore, Karnataka, India).

### Ethical considerations

Informed written consent was obtained from all the patients and took ethical approval from the central ethics committee of the NITTE (Deemed to be University).

### Sample collection and processing

Two milliliters of the peripheral venous blood in EDTA vacutainer was collected from each patient. PRAME was detected with real-time quantitative RT-qPCR in peripheral blood samples taken from patients and controls. Details of the method are described below.

### RNA isolation

According to manufacturer’s instructions, the RNA isolation was performed from EDTA anti-coagulated peripheral blood samples using an RNAiso Plus kit (Takara Bio Inc., Japan). RNA quantification was performed using a nano-drop spectrophotometer (Eppendorf).

### Complementary DNA (cDNA) synthesis

Complementary DNA (cDNA) synthesis was performed using the total RNA (500ng) through Primescript 1st-strand cDNA synthesis kit (Cat- RR037A, Takara) following the manufacturer’s instructions. The mixture is incubated at the following conditions: 37°C for 15 min and 85°C for 5 min in a thermocycler (Eppendorf). The synthesized cDNA was stored at −20°C (Thermo Fisher Scientific, USA) till further use.

### Reverse transcriptase-quantitative polymerase chain reaction (RT-qPCR)

Reverse transcriptase-quantitative polymerase chain reaction (RT-qPCR) was performed by SYBR Premix Ex Taq II (Cat- RR820A, Takara) by following the manufacturer’s instructions.

PCR master mix is prepared by adding 10 μl of SYBR Premix Ex Taq II (2x), 2 μl of cDNA template, 0.4 μl of ROX reference dye (50x), 0.8 μl each of forward and reverse primer of selected genes, and 6 μl distilled water. The experiment was performed using a real-time PCR system (AriaMx Real-Time PCR, Agilent, USA) following optimized thermocycler conditions, such as denaturation at 95°C for 10 min and 94°C for 30 s, annealing at 58–62°C (based on specific primers) for 60 s, elongation at 72°C for 90 s, and final extension at 72°C for 10 min with 40 cycles. Results were normalized to housekeeping gene as Beta-Actin (β-actin) and expressed as fold expression. Relative mRNA expression levels were calculated using the RQ=2−ΔΔCt method [12]. Healthy donors were recruited as controls. The following equations were used: Ct target gene−Ct βactin=ΔCt in acute leukemia samples and healthy donor samples and ΔCt sample−ΔCt mean of healthy donor=ΔΔCt. All samples were analyzed in triplicate and took the mean values were for further calculations. The products were subjected to 1.5% agarose gel electrophoresis (Fig. 2D) (Table 1).

**Table 1:** List of genes with primer sequences, size, and accession numbers

Sl. no	Gene name	Sequence (5′–3′)	Size (bp)	Accession No. (NCBI gene ID)
1	Beta-actin (β-actin)	F: TCCTTCCTGGGCATGGAG	207	NM_001101.2
		R: AGGAGGAGCAATGATCTTGATCTT		
2	PRAME	F: TGAAAATGGTGCAGCTGG	154	Reference No- [13]
		R: CGGGGAAATGTAGGAAGA		

### Statistical analysis

Numerical data were expressed as a mean and standard deviation; qualitative data were expressed as frequency and percentage. A paired-sample *t* test was conducted to determine the PRAME gene expression in cases and control samples. One-way ANOVA was conducted to compare between three or more groups. Spearman-rho method was used to test the correlation between numerical variables. The relationship between clinical parameters and PRAME gene expression were analyzed using chi-square and independent *t* test. The cutoff points for PRAME gene expression were generated using receiver operating curve (ROC); based on that, the cutoff values of overall survival (OS) were generated using Kaplan-Meier analysis. *P* < 0.05 was considered to be statistically significant. The data was analyzed using SPSS version 16.0 (Chicago, USA).

## Results

Out of 42 samples collected, 29 (69.4%) were males, and 13 (30.95%) were females, with a mean and standard deviation of 39.07 ± 22.22 years. Of which AML were of 22 (52.38%) cases, ALL were of 14 (33.33%) cases, and 6 (14.2%) cases which included other forms of leukemia. Cytogenetic analysis performed on 42 AL patients, displayed normal karyotype (*n*=18), abnormal karyotype (*n*=16), and culture failure (*n*=8). Hypodiploidy (*n*=7), hyperdiploidy (*n*=2), translocation (*n*=3), trisomy (*n*=2), and deletions (*n*=2) were reported abnormal cases.

### PRAME gene expression

**Fig. 1:**
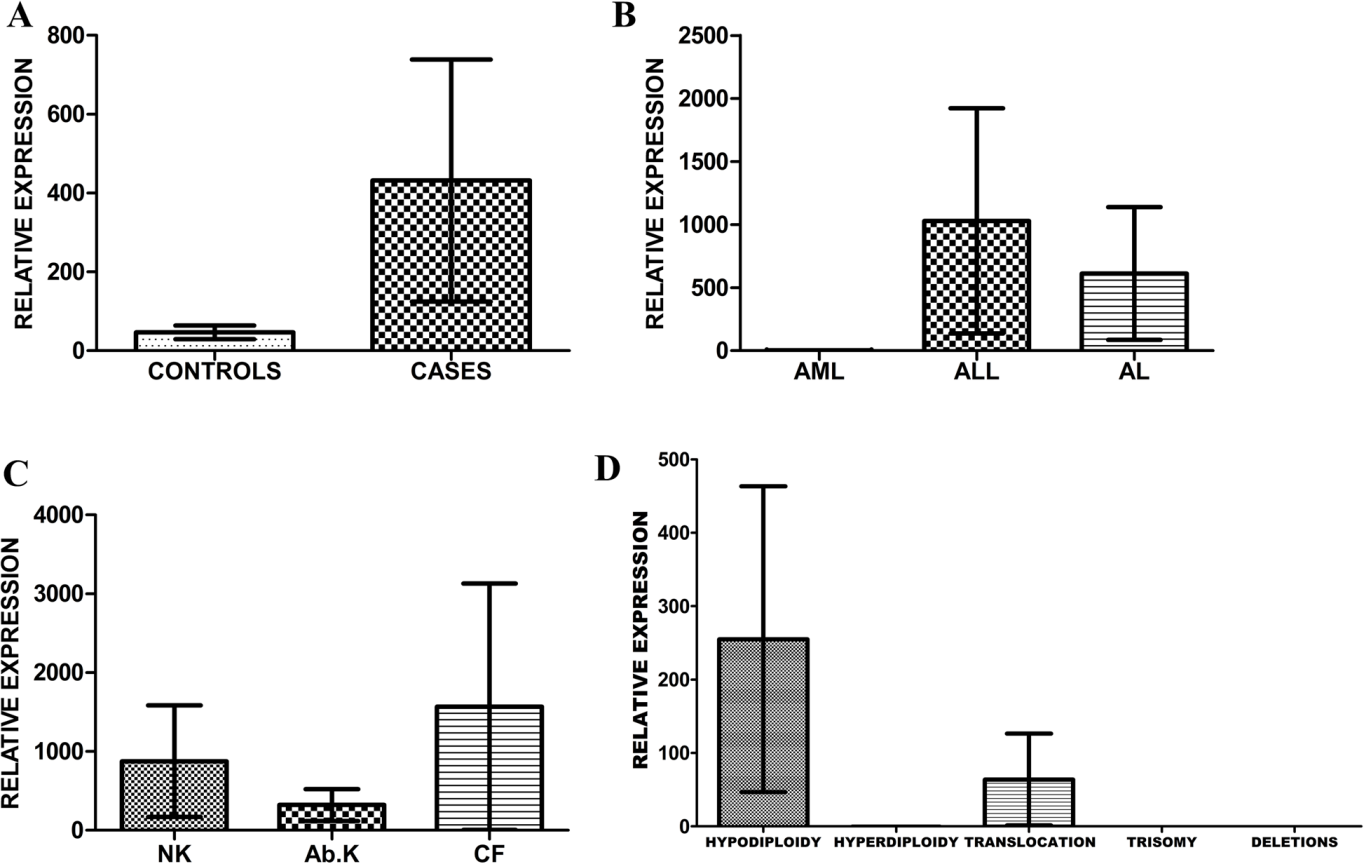
**A** Comparison of PRAME gene expression between patients and controls. **B** PRAME gene expression in acute leukemia classification. **C** PRAME gene expression based on cytogenetic analysis. **D** PRAME gene expression in various abnormalities of cytogenetic analysis

The comparison of PRAME gene expression was not statistically significant (*p*=0.3126) with a mean ± SD of 431.8 ± 307.4 (*n*=42) and 46.51 ± 17.19 (*n*=21) in cases and controls, respectively. PRAME gene expression was highly expressed in cases compared to controls. There was no statistical significance (*p*=0.3203) in PRAME gene expression between the classification of acute leukemia patients. Further, the cytogenetic analysis involving the normal karyotypes (NK), abnormal karyotypes (Ab. K), and culture failure (CF) also depicted statistically insignificance (*p*=0.5801). The PRAME gene expression in abnormal karyotype cases was found to be statistically not significant (*p***=**0.8507) among the hypodiploidy, hyperdiploidy, translocation, trisomy, and deletion cases (Table 2). The PRAME gene expression in all these groups was shown in (Fig. 1A–D).

**Table 2:** PRAME gene expression in acute leukemia patients

Categories	Subgroups	P value
PRAME gene expression	Controls	0.3811 (ns)
	Cases	
Classification of AL	AML	0.3203 (ns)
	ALL	
	AL	
Cytogenetic analysis	Normal karyotype (NK)	0.5801 (ns)
	Abnormal karyotype (Ab. K)	
	Culture failure (CF)	
Abnormalities in abnormal karyotypes	Hypodiploidy	0.8507 (ns)
	Hyperdiploidy	
	Translocation	
	Trisomy	
	Deletions

### Receiver operating curve (ROC) cutoff point analysis and Kaplan–Meier overall survival analysis

**Fig. 2:**
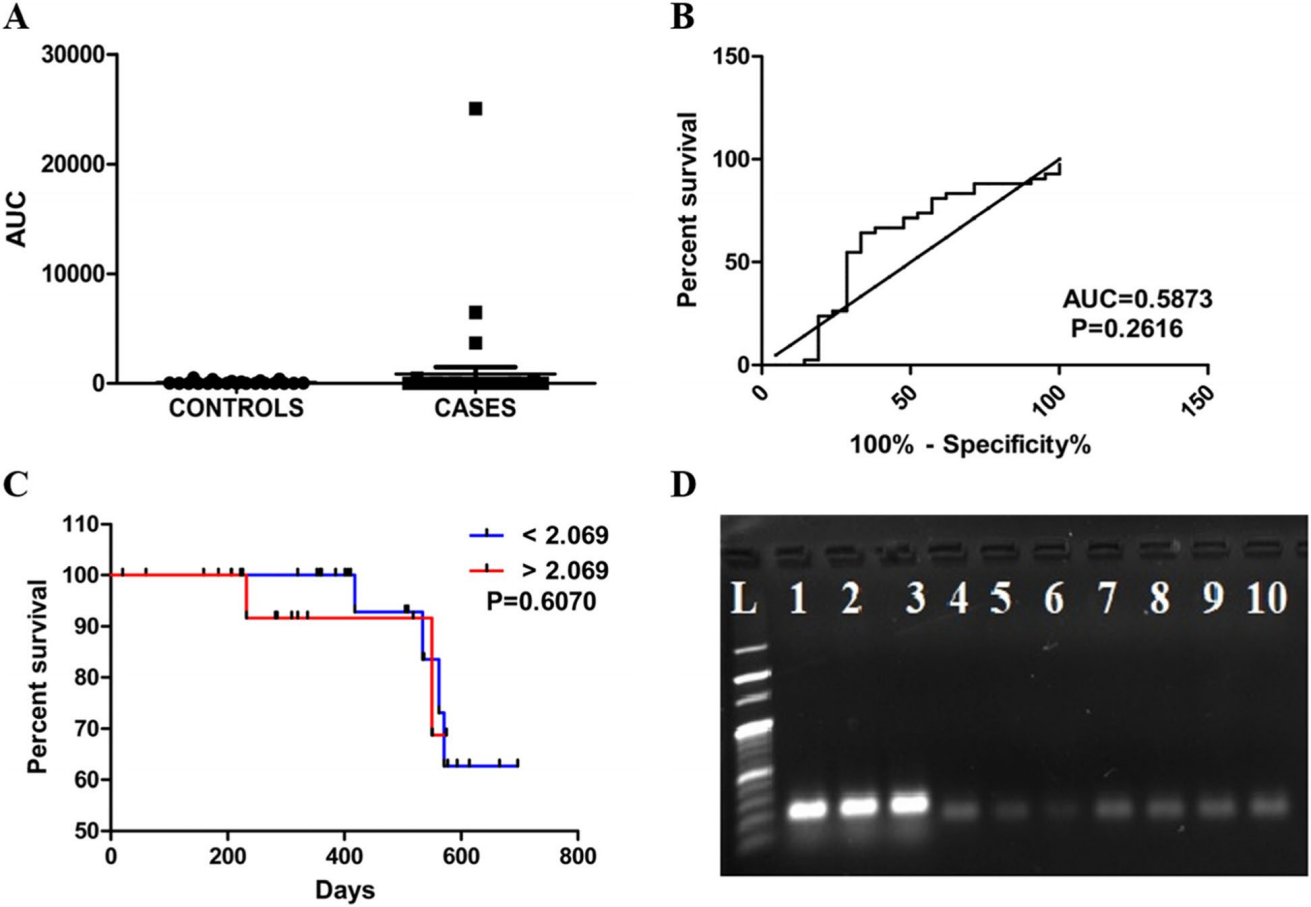
**A** Scatter plot graph with PRAME gene expression in cases and controls. **B** Receiver operating curve (ROC) for the PRAME gene obtained from the RT-qPCR. **C** Kaplan-Meier plot of overall survival in AML patients according to PRAME gene expression. **D** Electrophoresis representation of PRAME gene expression and B-actin expression in acute leukemia patients. *L*=100 bp DNA ladder; 1–3=B-actin gene; 4–10=PRAME gene

The cutoff value obtained was statistically not significant with an area under the curve (AUC=0.5873; *p*=0.2616 determined by ROC curve with the normalized β-Actin and relative expression of PRAME gene obtained from RT-qPCR. 2.069 was the cut-value obtained, which was further divided into higher and lesser value groups (Fig. 2A, B). Out of 42 patients, the PRAME gene expression was highly expressed in 27 (64.28%) patients and least expressed in 15 (35.71%) patients. The chloride, potassium and absolute lymphocyte count (ALC) was found to be statistically significant with *p*=0.0038**, *p*=0.0358*, and *p*=0.0216*, respectively, between all other clinical characteristics. The Kaplan–Meier analysis was performed in the 42 AL patients with subsequent data to investigate the association between PRAME expression and patient survival. Survival curves showed that AL patients with high PRAME expression had longer overall survival than those with low PRAME expression, which was statistically not significant (*p*=0.6070) with, hazard ratio= 0.6063, and 95% CI = 0.09008~4.081 (Fig. 2C).

### Correlation of clinical parameters with PRAME gene expression

The statistical evaluation was performed to study potential correlations between PRAME expression and clinical parameters. The results showed that PRAME gene expression was not significantly correlated with any of the mentioned parameters. The clinical and pathological data are summarized in Tables 3 and 4.

**Table 3:** Patients characteristics by ROC cutoff points in PRAME gene expression

Characteristics	PRAME < 2.609 (***n***=27)	PRAME > 2.609 (***n***=15)	***p*** value
Age (years)	35 (5–88)	41 (1–65)	0.4212
Gender: M/F	20/7 (74.07%/25.9%)	9/6 (60%/40%)	0.4880
WBC × 10^3^/L	34.3 (12–100)	30.4 (3.7–99.9)	0.5038
Lymphocyte %	65.7 (1.6–99.9)	67.1 (1.7–99.9)	0.4155
Hb (g/dL)	9.8 (5.9–15.3)	10.4 (6.6–15)	0.4157
ESR (mm/h)	68 (15–140)	78.5 (39–140)	0.4345
Total protein (g/dL)	6.9 (4.8–8.3)	6.9 (5.4–8.2)	0.3262
Albumin (g/dL)	3.9 (2.5–4.5)	3.6 (2.7–4.8)	0.2626
Globulin (g/dL)	3.2 (2.3–4.8)	3.3 (2.4–4.2)	0.9818
A/G ratio	1.1(0.7–1.8)	1.16 (0.7–1.8)	0.6092
Bilirubin direct	0.4 (0–2.4)	0.6 (0–2.2)	0.6246
Bilirubin indirect	0.43 (0.05–3.1)	0.5 (0.1–1.4)	0.2755
Bilirubin total (mg/dL)	0.9 (0.1–3.7)	1.3 (0.2–3.7)	0.7170
SGOT (U/L)	31 (11–62)	43 (18–76)	0.2026
SGPT (U/L)	29 (6.4–118)	33 (11–72)	0.3988
ALP (U/L)	88 (55–170)	102 (56–212)	0.2185
Chloride (mg/dL)	98 (1.1–120)	107 (89–133)	0.0038**
Sodium (mg/dL)	136 (118–177)	137 (120–157)	0.3402
Potassium (mg/dL)	3.7 (2.8–6)	4.2 (3–6.3)	0.0358*
Blood urea (mg/dL)	24 (8–101)	33 (9–144)	0.3772
TLC × 10^9^/L	23.1 (0.2–148)	33 (5.6–15)	0.7920
Creatinine	0.7 (0.2–1.7)	1.02 (0.2–2.3)	0.5621
Platelet count × 10^9^/L	148 (2–800)	226 (10–825)	0.7229
ALC × 10^9^/L	44.8 (1.7–97.7)	21.3 (9.1–74)	0.0216*
ANC × 10^9^/L	57 (70–571)	170 (187–605)	0.6847

*Hb* hemoglobulin, *ESR* erythrocyte sedimentation rate, *SGOT* Serum glutamic oxaloacetic transaminase, *SGPT* Serum glutamic pyruvic transaminase, *ALP* Alkaline phosphatases, *TLC* Total leucocyte count, *ALC* Absolute to lymphocyte ratio, *NLR* Neutrophil to lymphocyte ratio

Highlighted values are indicating statistical significance at *P* < 0.05 (Where, *P* < 0.05*, *P* < 0.01**)

**Table 4:** Correlation of clinical parameters with PRAME gene expression in acute leukemia patients

Clinical characteristics	PRAME gene expression	
	Rho (***ρ***)	***p*** value
Age (years)	−0.171	0.278
Hb (g/dL)	0.051	0.747
ESR (mm/h)	0.268	0.086
WBC × 10^3^/L	0.123	0.437
Lymphocyte %	−0.065	0.680
Total protein (g/dL)	−0.147	0.354
Albumin (g/dL)	−0.193	0.220
Globulin (g/dL)	−0.168	0.295
Bilirubin direct	−0.053	0.740
Bilirubin indirect	−0.036	0.821
Bilirubin total (mg/dL)	−0.059	0.709
SGOT (U/L)	0.060	0.704
SGPT (U/L)	0.021	0.893
ALP (U/L)	−0.074	0.639
Chloride (mg/dL)	0.062	0.698
Sodium (mg/dL)	0.076	0.634
Potassium (mg/dL)	−0.075	0.638
Glucose plasma (mg/dL)	0.105	0.510
Blood urea (mg/dL)	0.189	0.232
TLC × 10^9^/L	−0.045	0.778
Creatinine	0.022	0.890
Platelet count × 10^9^/L	−0.141	0.372
ALC × 10^9^/L	−0.131	0.408
ANC × 10^9^/L	−0.107	0.499

## Discussion

Acute leukemia is a hematologic disease that infiltrates the normal function of the bone marrow stem cells leading to the infiltration of malignant neoplastic cells in the disease progression, differentiation, and proliferation. It transforms effective cells into a mass of lymphoid and myeloid blasts in the bone marrow and peripheral blood. PRAME gene as a class of tumor antigen, having a varied expression in a different malignancy like chronic lymphoproliferative disorders, multiple myeloma, acute, and chronic leukemia’s. As a result of aberrant mRNA expression, the PRAME gene is subjected to quantitative analysis by real-time PCR for a better understanding of diagnosis, treatment monitoring, complete remission (CR), and minimal residual disease (MRD) of the disease by which we could know its role in association with other parameters like cytogenetic analysis as well [7, 9, 14].

Our study aimed to rule out the PRAME gene expression in AL patients and its prognostic value. PRAME gene expression was detected at a low level in peripheral blood samples obtained from normal healthy donors, while it was highly expressed in twenty-seven (64.28%) AL patients. PRAME gene was highly expressed in cases compared to controls as reported by many other study groups. The PRAME gene expression rate was 87% reported by Steger et al. and 74% by Baraka et al. and was higher when compared to our study. PRAME gene expression rate was 52.9% by A Spanaki et al., 42% by D Steinbach et al., 38.2% by Ding et al., 31.3% Abdel Malak et al., and 25% by Baren et al., were the reported cases, which was lesser compared to our study [5, 6, 9, 15–18]. These discrepancies of results between all these studies may be due to the various set of leukemic cells expressed in different population set-up (ethnicity), the number of patients recruited, and numerous age groups like a pediatric, adult, and elderly patients for the analysis used to detect the PRAME gene expression rate are the other causes. Several other studies have reported using other techniques like microarray, western blotting, flow cytometry, and quantitative RT-PCR that the PRAME gene has elevated expression in leukemic cells compared to normal healthy patients [19–21].

Among the different classification groups of acute leukemia in the present study, PRAME gene expression was highly expressed in ALL compared to other forms. Among the cytogenetics, the culture failure cases were highly expressed than the normal and abnormal karyotypes. Regarding the cytogenetic abnormalities, a statistically non-significant PRAME gene expression was highly reported in hypodiploidy (*n*=7) cases compared to other chromosomal abnormalities reported: translocation (*n*=3): *t*(15;17) in two cases and *t*(3;3) in one case; trisomy (*n*=2) 13 and 19 each in one case. Various studies reported that PRAME is correlated with chromosomal translocation like BCR-ABL *t*(9;22), PML-RARA *t*(15;17), and AML-ETO *t*(8;21) in which further investigation is needed to prove its mechanism at the molecular level as they have a significant role in the upregulation of PRAME gene.

Based on the overall survival analysis in the present study, the overexpression of the PRAME gene had longer survival rate than the least expression in which shorter overall survival rates were seen in patients. A study reported by EL Khateeb et al. showed patients had more prolonged relapse-free survival in PRAME gene expression. Overexpression of PRAME gene had a pattern of longer overall survival for the patients as published by Greiner et al. Fifteen months was the obtained median overall survival having a significant relationship between high PRAME gene expression and better overall survival conducted by Baraka et al. Steinbach and his colleagues conducted a study on pediatric patients where the rates of overall and disease-free survival in patients with high PRAME expression were higher than in patients with least expression. There was no correlation between the PRAME gene expression and progression-free survival in patients by Paydas et al. and co-workers. There was a similarity between the results published by several other studies and the current study with slight modifications in comparison [7, 9, 17, 22, 23].

As a result, the PRAME gene can be used as a potential marker for indicating a good prognosis in risk-adapted therapy. Further, to differentiate between the favorable and unfavorable prognosis, larger population-based studies are necessary to justify the role of relative PRAME gene expression using an ideal cutoff [17]. However, the present study depicted that there was no correlation with age, sex, and clinical parameters of the patients in PRAME gene expression with no statistical significance similar to studies conducted by Abdel Malak et al., Ding et al., and Payadas et al. The other studies conducted by Steinbach et al. had found a negative correlation between the PRAME expression with white bold cell count at diagnosis. Irrespective of the correlation between PRAME gene expression and all clinical parameters, the PRAME gene could be used as a potential marker for monitoring the malignancies with tumor-associated antigens (TAA) and leukemia-associated antigens (LAA) [16, 22].

## Conclusions

PRAME gene expression in AL patients was highly expressed with non-significant cytogenetic results than healthy individuals, consistent with the data or studies published globally. There were no other literature studies published in our Indian population to correlate results with the current research. Overexpression of the PRAME gene can be used as an excellent prognostic aspect for targeted-based immunotherapy if more studies come up with clinical trials. Limitations in our study include the lack of clinical, cytogenetic abnormalities, and flow cytometric analysis, which was impossible due to its retrospective nature, in addition to common financial constraints.

## Data Availability

Not applicable

## Abbreviations

AL: Acute leukemia
Ab.K: Abnormal karyotypes
AML: Acute myeloid leukemia
ALL: Acute lymphoblastic leukemia
AML-ETO: Acute myeloid leukemia-right twenty-one
*APML*: Acute promyelocytic leukemia
AUC: Area under curve
BCR-ABL: Breakpoint Cluster Region-Abelson Murine Leukemia
cDNA: Complementary DNA
CF: Culture failure
CR: Complete remission
CTL: Cytolytic T lymphocytes
EDTA: Ethylene diamine tetra acetic acid
HLA: Human leucocyte antigen
LAA: Leukemia-associated antigens
MRD: Minimal residual disease
NK: Normal karyotypes
OS: Overall survival
PML-RARα: Promyelocytic leukemia-retinoic acid receptor alpha
PLZF-RARα: Promyelocytic leukemia zinc finger-retinoic acid receptor alpha
PRAME: Preferentially expressed antigen of melanoma
RT-qPCR: Real-time quantitative polymerase chain reaction
*ROC*: Receiver operating curve
SPSS: Statistical package for social sciences
t: Translocation
TAA: Tumor-associated antigens
